# Application of ultrasound artificial intelligence in the differential diagnosis between benign and malignant breast lesions of BI-RADS 4A

**DOI:** 10.1186/s12885-020-07413-z

**Published:** 2020-10-02

**Authors:** Sihua Niu, Jianhua Huang, Jia Li, Xueling Liu, Dan Wang, Ruifang Zhang, Yingyan Wang, Huiming Shen, Min Qi, Yi Xiao, Mengyao Guan, Haiyan Liu, Diancheng Li, Feifei Liu, Xiuming Wang, Yu Xiong, Siqi Gao, Xue Wang, Jiaan Zhu

**Affiliations:** 1 Department of Ultrasound, Peking University People’s Hospital, Beijing, 100044 China; 2 School of Computer Science and Technology, Harbin Institute of Technology, Harbin, 150001 Heilongjiang Province China; 3 Department of Ultrasound, Southeast University Zhongda Hospital, Nanjing, 210009 Jiangsu Province China; 4 Department of Ultrasound, The First Affiliated Hospital of Guangxi University of Chinese Medicine, Nanning, 530023 Guangxi Zhuang Autonomous Region China; 5 Department of Ultrasound, Zhengzhou University First Affiliated Hospital, Zhengzhou, 450052 Henan Province China

**Keywords:** Artificial intelligence, Breast, BI-RADS 4A, Differential diagnosis

## Abstract

**Background:**

The classification of Breast Imaging Reporting and Data System 4A (BI-RADS 4A) lesions is mostly based on the personal experience of doctors and lacks specific and clear classification standards. The development of artificial intelligence (AI) provides a new method for BI-RADS categorisation. We analysed the ultrasonic morphological and texture characteristics of BI-RADS 4A benign and malignant lesions using AI, and these ultrasonic characteristics of BI-RADS 4A benign and malignant lesions were compared to examine the value of AI in the differential diagnosis of BI-RADS 4A benign and malignant lesions.

**Methods:**

A total of 206 lesions of BI-RADS 4A examined using ultrasonography were analysed retrospectively, including 174 benign lesions and 32 malignant lesions. All of the lesions were contoured manually, and the ultrasonic morphological and texture features of the lesions, such as circularity, height-to-width ratio, margin spicules, margin coarseness, margin indistinctness, margin lobulation, energy, entropy, grey mean, internal calcification and angle between the long axis of the lesion and skin, were calculated using grey level gradient co-occurrence matrix analysis. Differences between benign and malignant lesions of BI-RADS 4A were analysed.

**Results:**

Significant differences in margin lobulation, entropy, internal calcification and ALS were noted between the benign group and malignant group (*P* = 0.013, 0.045, 0.045, and 0.002, respectively). The malignant group had more margin lobulations and lower entropy compared with the benign group, and the benign group had more internal calcifications and a greater angle between the long axis of the lesion and skin compared with the malignant group. No significant differences in circularity, height-to-width ratio, margin spicules, margin coarseness, margin indistinctness, energy, and grey mean were noted between benign and malignant lesions.

**Conclusions:**

Compared with the naked eye, AI can reveal more subtle differences between benign and malignant BI-RADS 4A lesions. These results remind us carefully observation of the margin and the internal echo is of great significance. With the help of morphological and texture information provided by AI, doctors can make a more accurate judgment on such atypical benign and malignant lesions.

## Background

The Breast Imaging Reporting and Data System (BI-RADS) facilitates communications among radiologists, clinicians and patients via the use of standardised descriptions of lesions and reports, which greatly promotes the application of breast imaging in clinical practice. BI-RADS 4A lesions exhibit a low suspicion for malignancy of 2–10% and primarily include some atypical benign and malignant lesions [1]. The 2013 BI-RADS does not provide specific guidance for the sub-category of BI-RADS 4 lesions. The classification of these lesions is mostly based on the personal experience of doctors and lacks specific and clear classification standards. The large ultrasonic feature span of atypical benign and malignant lesions creates the possibility of misclassification in the BI-RADS 4A category.

The development of artificial intelligence (AI) provides a new method for BI-RADS classification [2]. AI can calculate the morphological and texture features of breast lesions in ultrasonic images and overcome the shortcomings of human visual observation [3–5]. At present, the application of AI in BI-RADS classification mainly focuses on the feasibility and accuracy of different AI procedures [6–10]. AI can achieve a classification level similar to that of radiologists [6, 9]. Through the quantitative study of BI-RADS classification features, some studies have reported morphological and textural features that are different between benign and malignant lesions. The shape, margin, internal echo and posterior echo of tumour can be used as the differential diagnosis points of benign and malignant lesions [6, 8, 11]. Some other studies focus on the differences in morphological and textural features among different BI-RADS categories or specific diseases, for example, triple-negative breast cancer and fibroadenoma [6, 12–14]. Studies investigating the application of AI between BI-RADS 4A benign and malignant lesions are limited. The present study analysed the ultrasonic morphological and texture characteristics of BI-RADS 4A benign and malignant lesions using AI and aimed at examining the value of AI in the differential diagnosis of BI-RADS 4A benign and malignant lesions.

## Methods

All of the patients were from Peking University People’s Hospital, Southeast University Zhongda Hospital, the First Affiliated Hospital of Guangxi University of Chinese Medicine and Zhengzhou University First Affiliated Hospital. The ethics committees of the four hospitals approved this study. Written informed consents were obtained from all participants. All the doctors participated in the ultrasonic examinations. All lesions diagnosed as BI-RADS 4A before surgery from January 2019 to December 2019 were collected and analysed retrospectively. According to the ACR BI-RADS® Atlas Fifth Edition, two doctors (SHN and XW) with more than 10 years’ experience in breast ultrasound diagnosis who were blind to the pathological results evaluated the suspicion for malignancy of all the lesions separately, and lesions with low suspicion for malignancy (2–10%) were classified as BI-RADS 4A.

The inclusion criteria were as follows: (1) lesions were classified as BI-RADS 4A by the two doctors finally; (2) the lesions were clear in grey-scale images without measurement labels and the sample window of colour Doppler; (3) lesions should be displayed within a high-frequency probe, and those less than 5 cm were included according to the width of high-frequency probes; (4) all lesions were surgically resected and pathologically diagnosed. The following exclusion criteria were employed: (1) lesions were displayed in colour Doppler ultrasound images; (2) measurement labels were present in grey scale images; (3) the transverse diameter of lesions exceeded the width of probes.

Among them, 194 lesions were both classified as BI-RADS 4A by the two doctors. Twelve cases with inconsistent classification were determined as BI-RADS 4A after discussion by the two doctors. Finally, 206 lesions were enrolled in our study.

The ultrasound instruments used in this study included Aixplorer (Supersonic Imagine, Aix-en-Provence, France), Aplio 500 (Toshiba Medical Systems, Otawara, Tochigi, Japan), and Logic E9 (GE Healthcare, Milwaukee, WI, USA) instruments.

The AI software used in this research was the breast ultrasound intelligent diagnosis system developed by the Harbin Institute of Technology. All lesions were manually contoured, and the region of interest (ROI) was calculated using grey gradient co-occurrence matrix analysis to obtain the morphological and texture features.

The morphological features included circularity, height-to-width ratio, margin spicules, margin coarseness, margin indistinctness, margin lobulation, internal calcification and angle between the long axis of the lesion and skin (ALS). The principles of these features were as follows:

1. Circularity

Circularity (Cir) described the similarity between tumours and circle, and it was calculated according to the following formula (1):

1 $$ Cir=\frac{C^2}{S} $$

C was the number of pixels in the tumour boundary, which was equivalent to the perimeter of the tumour, and S was the number of pixels contained in the tumour area, which could be regarded as the area of the tumour.

2 Height-to-width ratio

The height-to-width ratio (HWR) calculated the circumscribed rectangle of the tumour boundary first to obtain the height and width of the circumscribed rectangle and then calculated the ratio of the two using the formula (2):

2 $$ HWR=\frac{H}{W} $$

3 Margin spicules

The coordinates of the margin pixels (*x*_*i*_, *y*_*i*_) were set to coordinates in polar coordinates (*r*_*i*_, *θ*_*i*_) according to centroid coordinates (*x*_*0*_, *y*_*0*_). Then, the coordinates were rearranged clockwise (or anticlockwise). Then, Fourier transformation was performed, and the frequency spectrum data were obtained. The number of margin spicules (MS) was calculated according to the following formula (3):

3 $$ MS=\frac{\sum \limits_{\omega =0}^{\frac{\pi }{4}}R\left(\omega \right)}{\sum \limits_{\omega =\frac{\pi }{4}}^{\pi }R\left(\omega \right)} $$

4 Margin coarseness

Margin coarseness (MC) reflectd the degree of coarseness of tumour margin, which was given by Eq. (4):

4 $$ MC=\frac{1}{N}\sum \limits_{i=1}^N\mid {d}_i-{d}_{i+1}\mid $$

Here, *d*_*i*_ reflected the distance (in pixel units) of the *i*_*th*_ pixel on the boundary to the centroid coordinates of the tumour, and *d*_*i*_ was arranged and calculated according to the clockwise (or anticlockwise) order of the corresponding pixels on the boundary.

5 Margin indistinctness

The coarse boundary of tumour in the original grey-scale ultrasound image was calculated using a rough segmented ROI image, and tissue surrounding the tumour was regarded as the boundary area. The pixel gradient in horizontal and vertical directions of the boundary area was calculated using the Sobel operator, and the margin indistinctness (MI) was calculated according to the following formula (5):

5 $$ MI=\sum \limits_{i=1}^n\sum \limits_{j=1}^m\sqrt{d_x{\left(i,j\right)}^2+{d}_y{\left(i,j\right)}^2} $$

*M* and *n* represented the size of the image, and *d*_*x*_ and *d*_*y*_ represented the gradient in the horizontal and vertical directions of the pixel at the tumour boundary, respectively.

6 Margin lobulation

The coordinates of the margin pixels (*x*_*i*_*, y*_*i*_) were converted to coordinates in polar coordinates (*r*_*i*_*, θ*_*i*_) according to centroid coordinates (*x*_*0*_*, y*_*0*_). Here, *θ*_*i*_ was converted to the polar coordinate sequence (*r*_*1*_*, θ*_*1*_), (*r*_*2*_*, θ*_*2*_), *(r*_*3*_*, θ*_*3*_), (*r*_*i*_*, θ*_*i*_), (*r*_*n*_*, θ*_*n*_) according to the clockwise (or anticlockwise) order. The median filter of frame size 21 was used to reduce the influence of image noise, and the sequence was fitted with a polynomial of degree 20. The sum of the maximum and minimum points was obtained as the value of margin lobulation (ML) listed in the formula (6).
6

7 Internal calcification

First, the irrelevant region outside the tumour was set as zero pixels according to the coarse segmentation results, and the interior region of the tumour was binarized according to the mean grey value and the maximum value. Then, the binary image was processed by morphology expansion and corrosion to remove the interference pixels; finally, the number of connected regions of the white spots in the binary image was the number of internal calcifications in the image.

8 ALS

ALS *θ* described the angle between the tumour area and the horizontal direction. The ellipse fitting algorithm was used to fit the tumour boundary of ROI image, and the fitted ellipse centre, long axis, short axis, the positive angles of long axis and X axis were obtained. The following transformation was performed according to the formula (7):

7 $$ \Big\{{\displaystyle \begin{array}{c}\theta, 0\le \theta \le \frac{\pi }{2}\\ {}\pi -\theta, \frac{\pi }{2}\le \theta \le \pi \\ {}\theta -\pi, \pi \le \theta \le \frac{3\pi }{2}\\ {}2\pi -\theta, \frac{3\pi }{2}\le \theta \le 2\pi \end{array}} $$

Texture features included energy, entropy and grey mean. The number of pixels with a grey level of ***i*** and gradient of ***j*** in the gradient image simultaneously was the value of *H(i, j)*. Here, *H(i, j)* was normalised to obtain *P(i, j)*, and *P(i, j)* was used to calculate these texture features. The calculations of energy, entropy and grey mean were according to the formulas (8, 9 and 10), respectively:

1. Energy (E)

8 $$ E=\sum \limits_{i=1}^{L_s}\sum \limits_{j=1}^{L_g}P{\left(i,j\right)}^2 $$

2 Entropy (Ent)

9 $$ Ent=-\sum \limits_{i=1}^{L_s}\left(\sum \limits_{j=1}^{L_g}P\left(i,j\right)\ast \log \sum \limits_{j=1}^{L_g}P\left(i,j\right)\right) $$

3 Grey mean (GM)

10 $$ GM=\sum \limits_{i=1}^{L_s}i\ast \sum \limits_{j=1}^{L_g}P\left(i,j\right) $$

### Statistical analysis

The SPSS version 17.0 software package for Windows (IBM Corporation, Armonk, NY, USA) was used for data analyses. Descriptive statistics and frequencies were provided for circularity, height-to-width ratio, margin spicules, margin coarseness, margin indistinctness, margin lobulation, energy, entropy, grey mean, internal calcification and ALS, which were all nomal distribution. Means ± standard deviation were used to describe these features. Two independent samples t-test was used to compare two means in the sample. *P* < 0.05 indicated a statistically significant difference.

## Results

All of the 206 patients were female. All of the lesions were isolated. A total of 174 cases were benign. The median patient age was 39 years (range: 26–57 years), and the median lesion size was 1.6 cm (range: 0.6–4.2 cm). Thirty two cases were malignant. The median patient age was 43 years (range: 32–63 years), and the median lesion size was 1.3 cm (range: 0.8–2.5 cm). The pathological types of benign lesions and malignant lesions were presented in Table 1.

**Table 1:** Pathological types of benign lesions and malignant lesions

	Pathological types	*n*	%
Benign lesions	Adenosis	67	38.5
	Benign phyllodes tumour	6	3.4
	Fibroadenoma	68	39.1
	Inflammation	8	4.6
	Intraductal papilloma	20	11.5
	Complex cyst	5	2.9
Malignant lesions	Intraductal carcinoma in situ	12	37.5
	Intraductal papillary carcinoma	2	6.3
	Invasive ductal carcinoma	18	56.2

Data for the circularity, height-to-width ratio, margin spicules, margin coarseness, margin indistinctness, margin lobulation, energy, entropy, mean of grey level, internal calcification and ALS were presented in Table 2.

**Table 2:** Morphological and texture characteristics of benign and malignant groups

	Benign group	Malignant group	*P*
Circularity	23.62 ± 6.85	27.53 ± 13.45	0.240
Height-to-width ratio	0.59 ± 0.27	0.69 ± 0.16	0.343
Margin spicules	14.60 ± 9.40	14.67 ± 11.01	0.960
Margin coarseness (pixel)	2.78 ± 2.52	3.43 ± 3.00	0.420
Margin indistinctness (E6)	2.19 ± 9.03	2.23 ± 7.36	0.610
Margin lobulation	7.68 ± 2.55	9.58 ± 3.40	0.013
Energy	0.03 ± 0.02	0.04 ± 0.03	0.271
Entropy	2.34 ± 0.13	2.23 ± 0.20	0.045
Grey mean	0.52 ± 0.17	0.49 ± 0.28	0.957
Internal calcification	3.78 ± 4.29	2.53 ± 2.05	0.045
ALS (degree)	13.58 ± 18.42	6.32 ± 5.65	0.002

**Fig. 1:**
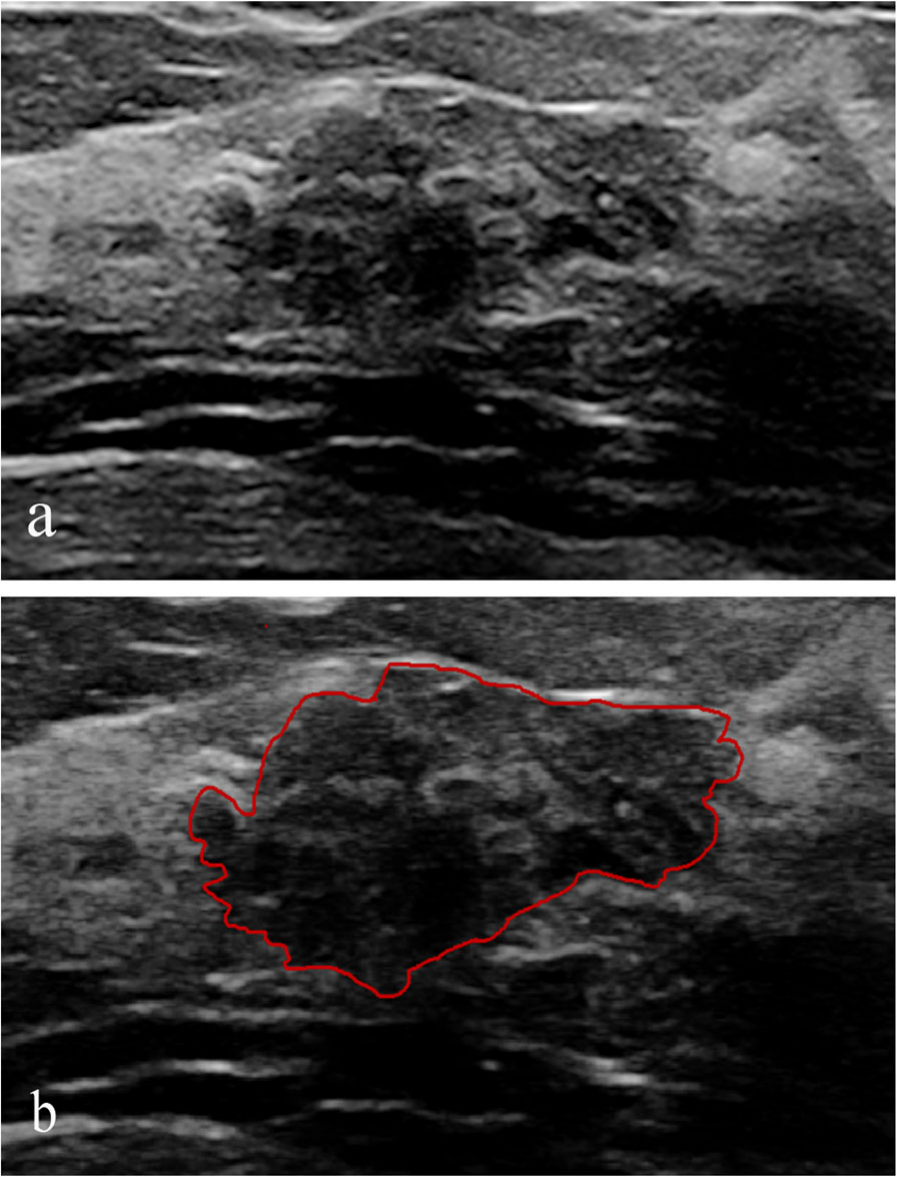
Intraductal carcinoma in situ classified as BI-RADS 4A. **a** Ultrasound revealed a solid hypoechoic mass with lobulation. **b** Image contoured manually

**Fig. 2:**
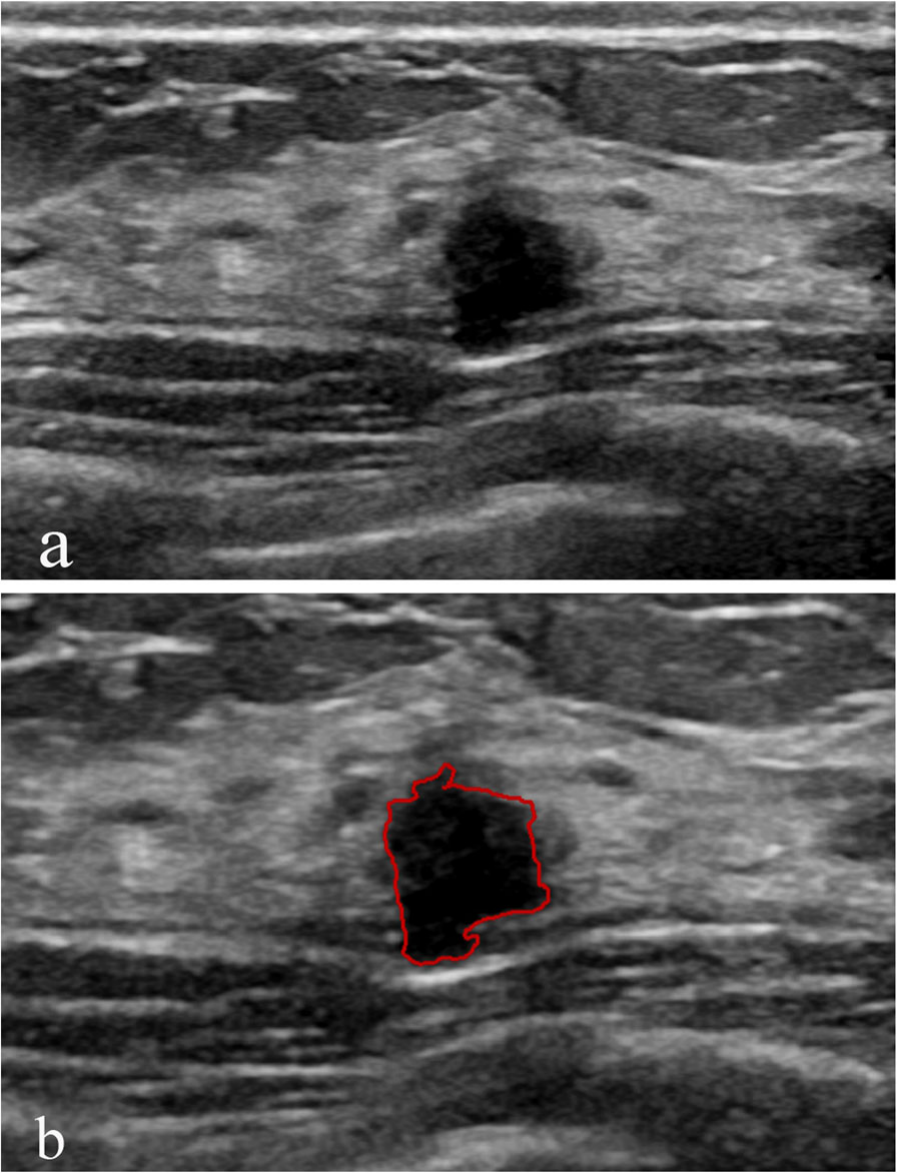
Adenosis classified as BI-RADS 4A. **a** Ultrasound revealed a solid irregular hypoechoic mass. **b** Image contoured manually

Statistically significant differences in margin lobulation, entropy, internal calcification and ALS were noted between the benign and malignant groups. The malignant group exhibited increased margin lobulation (Fig. 1) and lower entropy compared with the benign group, and the benign group had more internal calcifications and increased ALS compared with the malignant group (Fig. 2). No significant differences in circularity, height-to-width ratio, margin spicules, margin coarseness, margin indistinctness, energy, and grey mean were noted between the benign and malignant groups.

## Discussion

AI exhibits high accuracy in the diagnosis of breast lesions [15, 16]. AI significantly improves the diagnostic accuracy of doctors and improves the consistency among observers [7]. According to a study of BI-RADS 3 lesions, the computer-aided diagnosis system could correctly upgrade most malignant tumours misdiagnosed as Category 3 by doctors [12]. For Category 4A, AI also exhibited high diagnostic efficiency, and the classification accuracy of BI-RADS 4A can be greater than 0.9 [10, 14, 17].

Morphological and texture features are the main factors for AI diagnosis. According to the literatures, the use of morphological features and texture features is not limited to the diagnosis of benign and malignant diseases, and these features also help classify malignant tumour subtypes [13, 15, 17–19]. Entropy reflects the complexity and heterogeneous character of lesion texture. Larger entropy indicates more information contained in an image and greater uniformity of the pixel matrix of the image [20]. Compared to benign tumours, the internal components of malignant tumours are more complex. The different proportions of fibrous components, haemorrhage, necrosis, and calcification, result in a heterogeneous echo of malignant tumour. The increase in scattering media causes variation in backscattering, which reduces entropy. Therefore, compared with benign tumours, the entropy of malignant tumours is often reduced [20, 21]. Category 4A benign and malignant lesions are atypical benign and malignant lesions. Our study demonstrated that these atypical benign and malignant lesions were still consistent with previous studies [20, 21]. These findings suggest that careful observation of the internal echo of the lesions will help doctors improve the accuracy of naked eye diagnosis of difficult differentiations between benign and malignant tumours.

Category 4A benign and malignant lesions exhibited a significant difference in the number of margin lobulations. The biological behavior of the tumour determines the ultrasonic characteristics. The growth of cancer cells is not uniform and results in an irregular tumour morphology, which is lobulated. On the other hand, the ultrasonic characteristics of the lesions reflect the essential characteristics of the tumour, which is the basis for differentiating between benign and malignant lesions. Therefore, the characteristics of tumour margin are significant in the differentiation of atypical benign and malignant lesions, which is consistent with the literature [11].

Calcification can occur in both benign and malignant breast lesions. Most of the calcifications are benign, but a small portion is malignant [22]. Some benign tumours may have mucinous degeneration or hyaline degeneration with dystrophic calcification, which is occasionally difficult to distinguish from breast cancer calcification [23]. More calcifications were found in benign lesions in our research, which is consistent with early literatures [22, 24]. These characteristics increase the pathological uncertainty of benign lesions and make these lesions more atypical.

Most of the benign lesions grow in parallel, but atypical benign and malignant lesions may also exhibit unconventional characteristics. In this study, the ALS of benign lesions was larger than that of malignant lesions. In a sense, category 4A benign lesions are more like malignant lesions based on some ultrasound features. Category 4A malignant lesions exhibit fewer typical malignant signs, and some of their ultrasound features are more similar to those of benign lesions. These differences reflect the characteristics of category 4A lesions. The boundaries of some characteristics between category 4A benign and malignant lesions are indistinct or even inverted and deviate from the signs of typical benign and malignant lesions [25]. Difficulty in the differential diagnosis of the two groups causes the classification of benign lesions to be upgraded, whereas the classification of malignant lesions is downgraded.

Our study had some limitations. First, the size of our sample was relatively small. Future studies will include a larger number of cases. Second, in the aspect of intralesional calcification, we only studied the value of the number of calcification in the differential diagnosis of BI-RADS 4A benign and malignant lesions, but the significance of the size and shape of calcification in the differential diagnosis was not clear. Finally, this study was based on manually contoured images for quantitative analyses of ROI, which was different from other studies that focused on lesions that are automatically contoured by AI [26]. The present study did not evaluate the automatic identification efficiency for BI-RADS 4A lesions of our AI diagnosis system, and these aspects will be studied in the future.

## Conclusions

AI gives us a lot of inspiration. First of all, AI can find out the difference between benign and malignant lesions of BI-RADS 4A, which exceeds the recognition ability of human eyes. Secondly, AI reminds us we should carefully observe whether the lesions are more lobulated and whether the internal echo is more heterogeneous. Especially, the combination of the two features has higher diagnostic value. However, it need a large quantity of cases to determine the threshold of margin lobulation, entropy and internal calcification to diagnose malignant lesions of BI-RADS 4A, our cases are far from enough, especially for the malignant lesions. In the future, we will collect more lesions of BI-RADS 4A and summarize their characteristics so as to obtain a more accurate differential diagnosis threshold.

## Data Availability

The datasets used and/or analysed during the current study are available from the corresponding author on reasonable request.

## Abbreviations

BI-RADS: Breast Imaging Reporting and Data system
AI: Artificial intelligence
ROI: Region of interest
ALS: Angle between the long axis of the lesion and skin
Cir: Circularity
HWR: Height-to-width ratio
MS: Margin spicules
MC: Margin coarseness
MI: Margin indistinctness
ML: Margin lobulation
E: Energy
ENT: Entropy
GM: Grey mean
